# Driving the ambulance: an essential component of emergency medical services: an integrative review

**DOI:** 10.1186/s12873-021-00554-9

**Published:** 2021-12-18

**Authors:** Julia Becker, Karin Hugelius

**Affiliations:** 1 Institute for Disaster and Emergency Management, 141 69 Berlin, Germany; 2 Faculty of Medicine and Health, Örebro University, 70182 Örebro, Sweden

**Keywords:** Ambulance, Emergency medical services, Transition, Prehospital care, Driving, Safety

## Abstract

**Background:**

The transport of patients from one location to another is a fundamental part of emergency medical services. However, little interest has been shown in the actual driving of the ambulance. Therefore, this review aimed to investigate how the driving of the ambulance affects the patient and the medical care provided in an emergency medical situation.

**Methods:**

A systematic integrative review using both quantitative and qualitative designs based on 17 scientific papers published between 2011 and 2020 was conducted.

**Results:**

Ambulance driving, both the actual speed, driving pattern, navigation, and communication between the driver and the patient, influenced both the patient’s medical condition and the possibility of providing adequate care during the transport. The driving itself had an impact on prehospital time spent on the road, safety, comfort, and medical issues. The driver’s health and ability to manage stress caused by traffic, time pressure, sirens, and disturbing moments also significantly influenced ambulance transport safety.

**Conclusions:**

The driving of the ambulance had a potential effect on patient health, wellbeing, and safety. Therefore, driving should be considered an essential part of the medical care offered within emergency medical services, requiring specific skills and competence in both medicine, stress management, and risk approaches in addition to the technical skills of driving a vehicle. Further studies on the driving, environmental, and safety aspects of being transported in an ambulance are needed from a patient’s perspective.

## Background

Ambulance services worldwide provide out-of-hospital assessment, medical care, and treatment, and, if needed, transport of patients suffering injuries or illnesses. The organization of emergency medical services (EMS) and the ambulance personnel’s competence and formal roles differ globally. In some countries, the EMS are organized as part of fire brigades or rescue services; in others, they are organized as part of hospital emergency services. Ambulance personnel may consist of voluntary workers, firefighters, paramedics, nurses, or medical doctors [1]. Regardless of their formal titles, ambulance personnel need to assess, load, and move the patient safely and maintain a professional relationship with the patient and significant others [2]. In some countries, the duties of driving the ambulance and providing medical care are held by separate individuals. In other settings, a team of individuals is trained to fulfil either role [2]. Despite how the service is organized, being the patient of an ambulance service entails being dependent on the caregivers and their competence through all phases of prehospital care, including the alarm call, the pick-up scene, the transportation process, and finally, the arrival and handover to the hospital or other destination.

One fundamental of any ambulance service is to transport patients from point A to point B, most often during the provision of medical care of some kind. The transport itself may be lifesaving in time-critical conditions [3, 4]. However, little interest has been paid to the actual transport of the patient: in practice, how driving of the ambulance influences the patient’s condition and the medical care provided. The scientific interest in prehospital and ambulance care research has, for some time, focused on emergency medicine, traumatology, clinical assessment, non-conveyed patients, and referrals of ambulance patients [5]. Therefore, there is a need for this integrative review aimed at investigating how the driving of the ambulance affects the patient and care within emergency medical services.

## Methods

A systematic integrative review [6] was conducted.

### Search strategy

A systematic search in PubMed and Web of Science was conducted by the first author with the support of an academic librarian. The keywords used in each database were combinations of “ambulance driving,” “drive*,” “driving*,” “transport*,” “ambulance*,” “road safety,” “ambulance,” and “EMS.” The asterisk denoted truncation. The literature search was conducted on October 20, 2020, in PubMed and on October 21, 2020, in Web of Science.

**Fig. 1:**
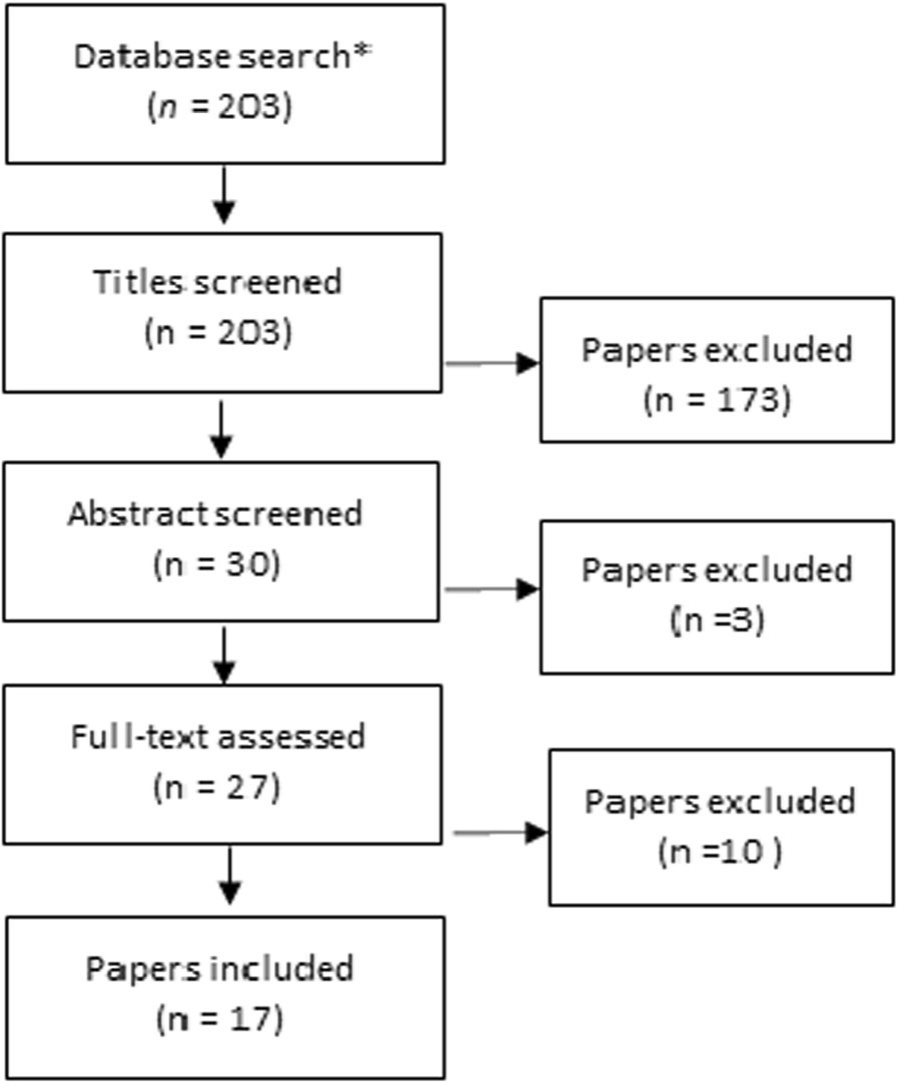
Overview of process to identify papers for review

The review used the following eligibility criteria for inclusion: [a] presented any aspect of the practice of driving an ambulance that influences the care provided; [b] published in English; [c] used a qualitative, quantitative, or mixed methods design; [d] published during the last ten years [2011–2020]. Exclusion criteria were [a] editorials, [b] papers covering helicopter, fixed wing, or military vehicle ambulance transport, [c] papers covering mass medevac transports, such as those using buses or similar vehicles, [d] papers focusing on technical kinematic aspects of ambulance collisions, [e] reviews, or [f] study protocols. The selection of papers to include was conducted by both authors using the Rayyan web application for systematic reviews [7]. If the authors had different views on whether a paper was relevant from a medical care perspective, the whole paper was read, and if there were still divergent estimations, the paper was included. An overview of the process to select papers for the review is shown in Fig. 1.

**Table 1:** Overview of literature search

	Search terms	Number of records
PubMed database	S1: [drive* OR driving* AND ambulance]	350
October 20, 2020 Language: English	S2: [transport*] AND [ambulance*]	2507
Publication dates: 2010–2020		
	Total	2857
Web of Science	S1: [drive* OR driving* AND ambulance]	162
October 21, 2020 Language: English	S2: [transport*] AND [ambulance*]	995
Publication dates: 2010–2020	Total	1157
	After removing duplicates	203
	Eligible for the selection process	203

### Quality appraisal

The CASP Cohort Study Checklist and the CASP Qualitative Studies Checklist [8] were used to assess the quality of the papers, depending on the methodology used. Under the integrative review methodology, a comprehensive appraisal of the overall quality of each study was made with the support from the CASP checklists [6]. Each study was first classified by both authors independently and thereafter discussed between the authors. If all criteria in the checklists were met, the study was considered to be of high quality. If most criteria, including a designated aim, a significant method used, or relevant conclusion drawn were met, the study was considered acceptable. Otherwise, the study was considered low quality. Studies assessed as acceptable or high quality were included.

### Analysis

The analysis conducted was inspired by the integrative review methodology [6]. The analysis was based on the extracted results of the included studies’ extracted results. First, the results relevant to this study’s aim were extracted. The extracted results were condensed and coded according to their content. All the codes were compared and organized to identify themes. In parallel, a mind map was used to illustrate the relationships between the themes. The analysis was initially made by the first author and continuously discussed between both authors until an agreed result emerged. Finally, a final result was formulated [6].

## Results

In total, 17 papers were included in the review (see Fig. 1). None of the studies identified in the search were excluded due to low quality, and no disagreements between the authors occurred during the quality appraisal process. The studies were conducted in Canada, Finland, the Netherlands, Korea, Spain, Sweden, the United Kingdom, and the United States. Both quantitative (11 studies) and qualitative (5 studies) methods were reviewed. Three studies were of experimental design, using simulators, for example, while the others relied on authentic ambulance transports or data drawn from real emergency calls.

**Table 2:** Overview of included papers and quality appraisal

Author/s	Year	Study focus	Country	Study design and method	Study population	Quality appraisal*
Beom et al	2018	Driving patterns and possibilities to conduct CPR during transport	Korea	Quantitative, analytic statistical analysis	Ambulance personnel (*n* = 48) and simulated driving patterns (*n* = 10)	High
Bui et al	2018	Driving pattern and behavior among ambulance drivers and impact on safety	USA	Quantitative, analytic statistical analysis	Driving data from 1.1 million km and ambulance crashes (*n* = 44)	High
Péculo-Carrasco et al	2020	Feelings of safety within the EMS	Spain	Qualitative, content analysis	Patients and ambulance personnel (*n* = 65)	Acceptable
Fleischmann	2013	Transportation time and the effects of navigation system support	USA	Quantitative analysis statistical analyses	Retrospective analysis of ambulance transports (*n* = 48,248)	High
Hoedemaker et al	2020	Timely transportation of percutaneous coronary intervention patients	The Nether- lands	Quantitative, analytic statistical analysis	Calculation of driving routes based on EMS records and postal codes	Acceptable
Jansson et al.	2020	Comparing transport time when using lights and sirens and no lights and sirens during interhospital transports	USA	Quantitative, analytic statistical analysis	Interhospital transports of critical patients (*n* = 5863)	High
Koski et al	2019	Safety risks and factors when caring within the EMS	Finland	Qualitative, inductive content analysis	Written statements of ambulance drivers (*n* = 44)	Acceptable
McDonald et al	2019	The impact of using lights and sirens during non-critical EMS transports	USA	Quantitative, analytic statistical analysis	Ambulance drivers (*n* = 80)	Acceptable
Missikpode et al	2018	Safety risks when driving with lights and siren	USA	Quantitative, quasi-induced exposure method	Emergency vehicle crashes (*n* = 2903)	High
Partridge et al	2020	Ambulance transport of infants with regards to the best way of driving to minimize the medical condition	UK	Quantitative, analytical statistical analysis	Mobile phone registration of EMS transports (*n* = 1700)	Acceptable
Petzäll et al	2011	Driving speed and pattern in relation to distances, the patient’s condition, and time saved	Sweden	Quantitative, analytic statistical analysis	Emergency high speed ambulance transportation in urban and rural areas (*n* = 30)	Acceptable
Suserud et al	2013	Caring for patients within the EMS during high speed transports	Sweden	Qualitative, content analysis	Individual interviews with ambulance personnel (*n* = 33)	Acceptable
Thézard et al	2019	Head and neck motion when transported in ambulances	Canada	Quantitative, analytic statistical analysis	Study persons (*n* = 18) and ambulance drivers (*n* = 12)	Acceptable
Tremblay et al	2020	Stress management in ambulance driving	Canada	Quantitative, analytic statistical analysis	Ambulance drivers (*n* = 17)	High
Venesoja et al	2020	Patients’ experiences of EMS safety	Finland	Qualitative, content analysis	Individual interviews with EMS patients (*n* = 21)	Acceptable
Watanabe et al	2019	Ambulance use of lights and sirens	USA	Quantitative, analytic statistical analysis	Emergency responses (*n* = 19 million)	High
Westerlund et al	2016	Driving pattern and occurrence of patients’ nausea	Sweden	Qualitative, content analysis	Individual interviews with ambulance nurses (*n* = 16)	Acceptable

* As determined by two of the authors

**Fig. 2:**
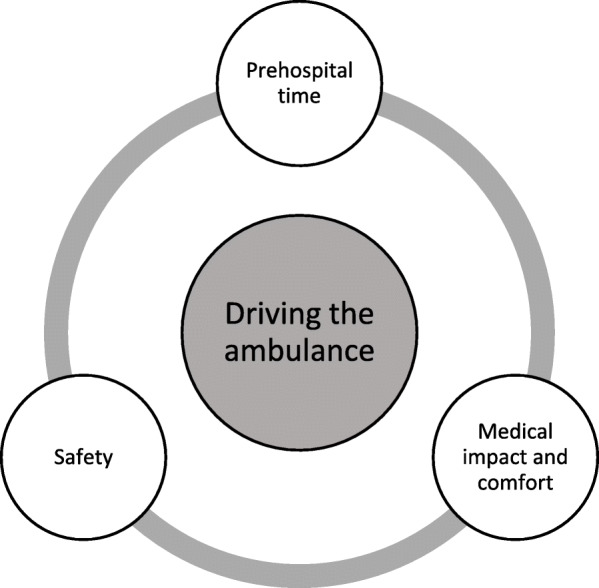
Perspectives on how driving the ambulance influenced the care within the EMS

The ambulance driving influenced the patient’s condition and the medical care from three perspectives, which were identified as themes in the analysis: prehospital time, medical impact and comfort, and safety. These perspectives interacted as essential parts of EMS care (see Fig. 2).

### Prehospital time

The physical movement of a patient from one place to another in the shortest time possible was an important aspect of the EMS care of patients in critical conditions [9–13]. Most often, driving the ambulance was focused on reducing the prehospital time. For time-critical transports, the ambulance driver had to choose the fastest route, which was not necessarily the shortest one. In addition, the route had to be suitable given the road conditions, weather, and other environmental factors. To decide what route to choose for the mission, technical devices such as navigation systems were used to support the ambulance driver [14] and estimate the prehospital time, time of day, rush hour, and to decide if there were need for lights and sirens [15]. To ease passing through traffic and reduce time spent on the road, lights and sirens were sometimes used to decrease total transport time, and the greatest time reduction was seen when conducting transports of critical patients and neonatal patients [13]. However, studies found that the use of sirens and lights should be balanced against an increased risk of crashes [10, 13, 16] and increasing stress for the driver, patients, and EMS personnel [17]. Therefore, the use of lights and sirens should be reserved for patients most likely to benefit from decreased transport time [13, 17].

### Medical impact and comfort

The way the ambulance was driven (e.g., accelerating, decelerating, turning, and adjusting to speed bumps and other roadblocks) and the quality of the road had direct impacts on the patient’s medical condition. Vibrations, hard or sudden movements, braking, or acceleration caused injuries or severed the medical conditions [14, 18, 19]. Noise did not only directly affect the patient’s condition [14] but also cause discomfort [12]. Ambulance acceleration was found to cause movement in the head and neck of patients, regardless of measures such as immobilizing the patient on a spine board, among others [18]. Careless driving or a road with many turns could cause motion sickness and/or nausea that also increased the risk of aspiration [19]. Another impact of the driving of the ambulance was that it could determine the kind of medical interventions that could be conducted. For example, the possibility of conducting cardiopulmonary resuscitation (CPR) and the quality thereof were greatly affected by driving patterns such as speeding, turning, or crossing road bumps [11]. To gain trust and make the patient feel comfortable during the transport, information provided by both the driver and EMS personnel during the actual transport increased the patient’s experiences wellbeing and overall trust of the EMS [20].

### Safety

Several factors influenced the safety of all people transported in the ambulance. One obvious risk was crashes. High speeds, regardless of the actual impact of time on the patient’s condition, increased the risk of crashes [21]. However, the driving pattern, in addition to the speed itself, had an effect on safety. Using an aggressive driving style with harsh braking and excessive speeding was associated with an increased risk of crashes [22]. The impact of using sirens and lights was uncertain from a safety perspective. In one study, such use was associated with an increased risk of crashing [10] and caused physical stress reactions in the driver that increased the risk of accidents [17]. In another study, however, no increased risk was observed [16]. By using seatbelts or stretcher belts and securing all loose items on board, one study found that the consequences of a crash could be mitigated [12]. In addition, such a procedure instilled a feeling of being safe among the patients [12, 23]. The perceived driving skills of the ambulance driver also increased the patient’s feeling of being safe [23].

Driving the ambulance was associated with stress due to time pressure [24], traffic situations [24, 25], poor visibility, and disturbing moments that distracted from focusing on driving [25]. Experienced drivers managed the stress better than inexperienced drivers or ordinary car drivers [24] and managing stress while driving an ambulance was found to be a skill possible to develop [24]. However, drivers with previous health conditions such as high blood pressure showed more physical stress reactions and had an increased risk of collision, regardless of their experience [24].

## Discussion

The results found that ambulance driving influenced both the patient’s medical condition and the EMS care of the patient by having a potential effect on prehospital time spent on the road, safety, and medical and comfort factors. Therefore, driving the ambulance itself needs to be considered an essential part of care within emergency medical services.

Prehospital times are important to reduce mortality, especially in time-critical conditions such as severe trauma [3]. However, all prehospital time is not the same. Studies have indicated that it may not be the total prehospital time, but time spent on the scene, that has the biggest impact on mortality [26]. Therefore, it seems reasonable to reflect on where time should be saved in the prehospital process to reduce total prehospital time. As reflected in many of the included studies, the actual transportation time must be weighed not only against increased safety risks but also against possible effects on the patient’s medical condition and the possibilities of providing adequate medical care during transportation. By reducing time spent on the scene, actual transport could be performed at a slower speed, thereby reducing risks and discomfort for all involved without a negative impact on the patient’s survival and wellbeing. Although this hypothesis cannot be concluded from this review, it is a potential research question.

The use of lights and sirens when transporting a time-critical patient has been debated for many years, mainly from a traffic safety or time-saving perspective. On one hand, using lights and sirens may reduce time spent on the road [27]. On the other hand, lights and sirens have also been found to increase stress among patients transported in an ambulance [28]. Therefore, the use of lights and siren must be a carefully considered intervention where the comfort and safety of the patient is balanced against the potential benefits of rapid transport. In addition, vibration, general noise, change in temperature, restricted space in the ambulance, and unexpected events influence the clinical condition of critically ill intensive care patients during interhospital transport [29]. It seems reasonable that the same effects also apply to non-critical patients. However, this review indicates that negative influences from lights and sirens, as well as other environmental aspects, might be mitigated or enlarged by the way the ambulance is driven, thereby confirming the importance of integrating the actual driving within the overall emergency medical services context of care. None of the studies included focused on how the technical design of the ambulance or the patient stretcher influenced the experience of being transported in the ambulance. However, since environmental aspects seem to be of importance for both the medical condition and safety of the patient, such studies are needed.

Several studies have focused on the safety aspects of ambulance transports. Patient safety usually refers to the absence of preventable harm to a patient during the health care process and the reduction of the risk of unnecessary harm associated with health care to an acceptable minimum [30]. The occurrence of ambulance crashes causes both injuries and death of patients and EMS personnel. The risk of transportation-related injury for EMS personnel has been reported to be about five times higher than the national average [31]. Training, risk awareness, and management by the drivers may be effective approaches in reducing the occurrence of emergency vehicle accidents [32]. Time pressure, multitasking activities, long shift hours, and the use of lights and sirens have been identified as risk factors for emergency vehicle crashes [33]. No studies were identified that explored ambulance crashes from the patients’ or relatives’ perspective. Being safe is not always the same as feeling safe. Information, communication, and a feeling of being accounted for have been suggested as tools to increase patients’ feelings of being safe [34]. In this study, being fastened with the seat or stretcher belt was found to increase the patient’s feeling of safety. Given the special context of being transported in an ambulance, further research on how to, in practice, promote feelings of safety in the patient (and significant others) is of interest.

Given the general idea of EMS, it was surprising that studies about the driving of the ambulance from a patient’s perspective were limited. However, this is in line with previous findings that caring science research with an explicit patient perspective within emergency medical services is limited [5]. It should be remembered that the transport of a person is not just a physical movement, as in moving a patient from the scene of an accident or from home to a hospital. It also entails a personal process. Only one of the studies touched on this phenomenon, in which patients described the value of being informed about what was going to happen next during the actual ambulance transport [20]. Transition means to pass from one condition, action, or place to another [35]. The first phase of a transition process is the movement *from* a situation or a physical place. This phase includes a *separation*. In the next phase, the person is *in the middle of something* but has not yet ended up in a new state. Being transported in an ambulance entails several impressions that have an impact on both medical condition and wellbeing, but it is also the middle phase of the transition process. Since disorientation, disintegration, and discovery are attributed to this phase [35], the ability to establish timely, safe, and comfortable transport that does not negatively affect the patient’s medical condition is essential. The third phase, a *new beginning*, where the person tries to incorporate new identities, behavioural patterns, and new ways of dealing with themselves and others in a new physical environment [35], can be seen as the arrival at the emergency department or new hospital ward. Exploring the actual transport of a patient within the EMS process from a transition perspective as well as an EMS caring perspective may promote increased wellbeing and comfort.

Differences in how ambulance services are organized and the large variation in the ambulance personnel’s general competence make it difficult to generalize concepts or standards for the driving of an ambulance. Drivers can be dedicated team members with limited medical education, volunteers, firefighters, or people with other professional backgrounds. In some contexts, the driver is one member of an equally trained ambulance crew, and the driving duty is shared within the team. Despite these differences, this study has pointed out that the actual transport and the way the ambulance is being driven is an important part of care provided within the EMS. Sufficient teamwork between the person driving the ambulance and the personnel providing direct care to the patient seems essential. However, no study in this review mentioned this aspect. That could either indicate a lack of understanding of the role of driving among medical and nursing researchers, or it could indicate that this is already a well-established way of working within the ambulance team that does not require further scientific attention. However, since few studies were found, the first option seems more likely. Likewise, studies did not reflect the relative risks or benefits of transporting patients, in particular during circumstances that might increase the risks, such as bad weather, snowstorms or bad road conditions. Given the increased rate of interhospital transports, such risks should be part of the medical risk assessment when transferring a patient. It seems reasonable to consider the actual driving of the ambulance as a specific skill and competence among EMS services, essential to providing high-quality and safe care. It can be suggested that driving the ambulance requires knowledge of how the actual driving affects the medical conditions and wellbeing of patients as well as safety aspects for patients and the EMS team. The driver must also have technical driving skills. Furthermore, personal competences such as stress management and the ability to manage complex traffic are important, as is the physical health and wellbeing of the driver.

### Limitations

This study was based on scientific papers published within the last ten years. With a longer timeframe, more papers would likely have been identified. However, most of the included papers were published in 2019 or 2020, indicating a growing interest in researching aspects of ambulance driving. Without standardized search terms for this topic, the literature search was based on keywords. This might have limited the possibilities of identifying relevant studies. Finally, as in every review, it cannot be assumed that the search or selection process identified all papers of interest.

## Conclusion

Driving the ambulance had a potential effect on patient health, wellbeing, and safety. Therefore, driving should be considered an essential part of medical care within EMS, requiring specific skills and competence in both medicine, stress management, and risk approaches in addition to the technical skills of driving a vehicle. Further studies are needed on driving, the environment, and the safety aspects of being transported in an ambulance.

Driving the ambulance requires specific medical, navigation, and stress management, and has a potential impact on a patient’s medical condition, wellbeing, and safety. Therefore, driving the ambulance should be considered an essential part of the care provided within the EMS, and the ambulance driver should be considered a team member. Given that the actual transport itself may have a significant impact on the patient’s medical condition, a balanced risk-benefit assessment should be conducted before all ambulance transports.

## Data Availability

Not applicable.

## Abbreviations

EMS: Emergency Medical Services
CASP: Critical Appraisal Skills Programme
